# Regulatory effect of miR-142-5p on inflammatory process of sepsis by targeting *CXCL8*

**DOI:** 10.1186/s41065-025-00567-5

**Published:** 2025-10-09

**Authors:** Ling Wu, Qiqi Shen, Xue Yu, Youfu Li, Dongcai Feng

**Affiliations:** 1 Intensive Care Unit, Nanjing Jiangning Hospital, Nanjing, 211100 China; 2 Department of Emergency, Shanghai Ninth People’s Hospital, Shanghai JiaoTong University School of Medicine, Shanghai, 200011 China; 3 Department of Infectious Diseases, Zhengzhou Central Hospital Affiliated to Zhengzhou University, Zhengzhou, 450000 China; 4 Department of Critical Care Medicine, Yiyang Central Hospital, Yiyang, 413000 China; 5 Cardiology Department, The Third Xiangya Hospital of Central South University, No.138 Tongzipo Road, Yuelu District, Hexi, Changsha City, 410006 Hunan Province China

**Keywords:** miR-142-5p, *CXCL8*, Sepsis, Molecular diagnostic marker, Targeted regulation

## Abstract

**Background:**

Sepsis is a life-threatening systemic inflammatory response triggered by infection. The rapid progression of the disease necessitates early diagnosis and precise intervention, making the identification of reliable biomarkers and therapeutic targets crucial for improving clinical outcomes and reducing sepsis-related mortality.

**Aim:**

Exploring miR-142-5p as a novel diagnostic biomarker for sepsis and its therapeutic potential via targeting *CXCL8*.

**Methods:**

The expression levels of inflammatory factors (IL-6, TNF-α, IL-1β) and miR-142-5p in the serum of patients with sepsis and healthy controls were detected by ELISA and qPCR methods respectively. The diagnostic potential of miR-142-5p was evaluated using Pearson correlation, ROC curve, and logistic regression analyses. Bioinformatic prediction and dual-luciferase assays identified *CXCL8* as a target, while LPS-induced models and cell transfection experiments investigated miR-142-5p’s therapeutic effects through *CXCL8* regulation.

**Results:**

Sepsis patients exhibited significantly decreased miR-142-5p expression, inversely correlating with inflammatory markers (IL-6, TNF-α, IL-1β). ROC analysis showed excellent diagnostic value (AUC = 0.917). Pearson correlations indicated significant clinical associations, while logistic regression identified miR-142-5p as an independent protective factor (HR = 0.498, 95% CI:0.282–0.882, *P* = 0.017). LPS models confirmed miR-142-5p’s anti-inflammatory effects through cytokine suppression, with knockdown showing opposite effects. Mechanistically, dual-luciferase and transfection assays verified *CXCL8* as a direct target mediating these effects.

**Conclusion:**

miR-142-5p may alleviate sepsis by targeting *CXCL8*-mediated inflammation, suggesting its potential as a diagnostic biomarker and therapeutic target.

## Introduction

Sepsis represents a life-threatening systemic inflammation caused by infection. It involves hyperactive immune responses and dysregulated cytokine release [1]. During sepsis, pathogen invasion triggers activation of both NF-κB and MAPK pathways. This interaction triggers immune cells to overproduce critical inflammatory mediators TNF-α, IL-1β, and IL-6 - establishing a pro-inflammatory cascade [2]. The excessive release of these inflammatory cytokines not only causes systemic inflammatory responses but also further leads to thrombosis and organ failure [3, 4]. As one of the most critical clinical conditions, sepsis has a mortality rate as high as 30–45% and represents a major cause of death in hospitalized patients [5]. The pathophysiological progression of sepsis is extremely rapid, with mortality increasing by 10% for each hour of delayed treatment [6]. Early recognition and precise treatment are therefore the core principles of sepsis management. Identifying sepsis biomarkers and therapeutic targets is now a key research priority.

Recent advances in anti-inflammatory strategies have highlighted two pivotal mechanisms: inflammasome signaling and ER-stress pathways. Notably, natural products like Terminalia chebula extracts demonstrate potent sepsis-modulating effects by simultaneously suppressing NLRP3 inflammasome activation and ER-stress-induced NF-κB/MAPK hyperactivation [7]. Its active components (e.g., 1,3,6-Trigalloylglucose and chebulinic acid) specifically target these pathways [8], while neochebulinic acid further exhibits antimicrobial synergy against gram-negative pathogens [9] –a feature particularly relevant to LPS-driven sepsis models. However, such small-molecule approaches often face challenges of off-target effects and limited bioavailability, creating a need for more precise interventions.

In contrast to small-molecule drugs, miRNAs offer inherent advantages in precision targeting. As 18–25 nucleotides (nt) endogenous non-coding RNAs, miRNAs fine-tune protein expression through sequence-specific interactions with 3’-UTR regions, leading to mRNA degradation or translational repression [10, 11]. Among them, miR-142-5p has been identified as an important inflammatory modulator. Studies demonstrate its involvement in autoimmune dacryoadenitis via *RC3H1* regulation [12], while both clinical and experimental data reveal its marked downregulation in sepsis and related kidney injury, highlighting its diagnostic potential [13].

The C-X-C Motif Chemokine Ligand 8 (*CXCL8*) gene encodes the pro-inflammatory cytokine interleukin-8 (IL-8) [14]. Studies indicate that *CXCL8* expression is significantly upregulated during sepsis, and its level changes are closely associated with the severity of sepsis-associated encephalopathy (SAE), highlighting its potential value as a diagnostic biomarker for SAE [15]. *CXCL8* may protect against sepsis-associated acute kidney injury through NF-κB pathway activation [16]. This miR-142-5p/*CXCL8* axis may complement existing anti-inflammatory strategies by providing multi-level control over the same NF-κB/MAPK nodes targeted by natural products, while avoiding their pharmacokinetic limitations.

However, whether *CXCL8* and miR-142-5p cooperatively regulate sepsis-induced inflammation through direct targeting remains poorly investigated. Therefore, this study aims to systematically elucidate the expression characteristics and clinical significance of miR-142-5p in septic patients, thoroughly investigate the targeting relationship between miR-142-5p and *CXCL8*, and reveal the regulatory mechanism of the miR-142-5p/*CXCL8* molecular axis in the inflammatory response of sepsis by integrating multi-level research approaches, including clinical sample analysis, cell experiments, and animal models. These findings may contribute to the development of novel diagnostic approaches, while the mechanistic insights could inform future therapeutic strategies pending further validation.

## Materials and methods

### Study participants

The study cohort comprised 220 participants, with equal distribution (*n* = 110 per group) between sepsis patients and age-matched healthy controls. All participants were recruited from The Third Xiangya Hospital of Central South University between March 2024 and October 2024. Sepsis patients met the following inclusion criteria: (1) fulfilled sepsis-3 diagnostic criteria (SOFA score ≥ 2 points due to infection); (2) age ≥ 18 years. Controls were selected from healthy individuals undergoing routine physical examinations during the same period. Exclusion criteria for all subjects included: (1) death within 24 h of admission; (2) comorbid malignancies or severe chronic diseases (e.g., cardiovascular diseases, hepatic/renal insufficiency); (3) those who have suffered from autoimmune diseases or have received immunosuppressive therapy within three months; (4) pregnancy or lactation.

The research protocol received ethical approval from the Ethics Committee of The Third Xiangya Hospital of Central South University and complied with Helsinki Declaration guidelines. All the subjects signed the informed consent form. All sepsis patients received standard care according to the Surviving Sepsis Campaign (SSC) guidelines and relevant professional practice standards during the study period.

### Clinical data and sample collection

Demographic and clinical characteristics including age, sex, body mass index (BMI), albumin, white blood cells (WBC), serum creatinine (Scr), C-reactive protein (CRP), and procalcitonin (PCT) were systematically collected for all participants. Complete data are presented in Table 1.

**Table 1:** Clinical characteristics of the subjects

Variable	Control (*n* = 110)	Sepsis (*n* = 110)	*P* value
Age (years)	52.60 ± 7.05	53.48 ± 8.92	0.417
Sex			0.344
male	58	65	
female	52	45	
BMI (kg/m^2^)	25.47 ± 3.14	25.02 ± 3.18	0.292
Albumin (g/L)	40.82 ± 4.35	27.65 ± 5.92	<0.001***
WBC (×10^9^/L)	6.16 ± 1.35	13.82 ± 3.81	<0.001***
Scr (µmol/L)	76.55 ± 18.69	128.72 ± 32.91	<0.001***
CRP (mg/L)	5.89 ± 1.87	72.19 ± 20.17	<0.001***
PCT (ng/mL)	0.05 ± 0.03	6.88 ± 3.40	<0.001***
APACHE II score	-	13.73 ± 5.17	-

BMI, body mass index; WBC, white blood cells; Scr, serum creatinine; CRP, C-reactive protein; PCT, procalcitonin; APACHE II, acute pathology and chronic health evaluation II. *** *P*<0.001

During clinical consultations, fasting venous blood (5 ml) was collected into serum separation tubes and centrifuged (4 °C, 3000 rpm, 10 min) using a refrigerated centrifuge.

### Cell culture and model induction

This study utilized the THP-1 human monocyte cell line (Solarbio, China) to establish an in vitro inflammatory model. The cells were cultured in RMIP-1640 medium containing 10% fetal bovine serum (Solarbio, China) and 1% penicillin-streptomycin (Biyantian, China), and placed in a 37 °C, 5% CO₂ incubator. Change the culture medium every 72 h. The experimental grouping design is as follows: (1) Normal culture control group; (2) LPS stimulation group: THP-1 cells were treated with 1 µg/ml LPS (Thermo Fisher Scientific, USA) at 37 °C for 24 h to establish a sepsis pathological model.

### Cell transfection

The THP-1 monocytic cell line was selected for its homogeneous genetic background and well-characterized inflammatory responses, avoiding confounding effects from intercellular heterogeneity present in mixed leukocyte populations. This aligns with single-cell transcriptomic studies demonstrating the precision of clonal cell lines for pathway-specific investigations [17], particularly in sepsis-related inflammation where cellular uniformity enhances data reproducibility [18].

The well-cultured THP-1 cells were systematically assigned to five experimental groups: (1) negative control mimic group; (2) miR-142-5p mimic group; (3) miR-142-5p inhibitor group; (4) *CXCL8* overexpression plasmid group; (5) co-transfection group (miR-142-5p mimic combined with *CXCL8* plasmid). The cell transfection experiment strictly followed the product manual and was completed using Lipofectamine™ 3000 (Thermo Fisher Scientific, USA) transfection reagent. Following a 48-hour incubation period post-transfection, subsequent experimental analyses were performed.

### qRT-PCR

Total RNA was extracted from serum and cells using TRIzol reagent (Invitrogen, USA), followed by cDNA synthesis with the PrimeScript RT kit (Takara, Japan). qRT-PCR was performed using SYBR Premix Ex Taq (Takara, USA) on a real-time PCR system (Thermo Fisher Scientific, USA) with the following cycling parameters: 95 °C for 30 s; 40 cycles of 95 °C for 5 s and 60 °C for 30 s. U6 and HPRT1 were used as reference genes, with primers (Table 2) meeting stringent criteria (GC content 40–60%, Tm 60 ± 2 °C, amplicon size 80–150 bp) and validated by melt curve analysis (single peak) and agarose gel electrophoresis (single band). Gene expression was calculated using the 2^−ΔΔCt^ method.

**Table 2:** Primer sequences for real-time quantitative polymerase chain reaction

Gene		Sequences (5´-3´)
miR-142-5p	Forward	GCCGAGCATAAAGTAGAAA
	Reverse	GTGCAGGGTCCGAGGT
CXCL8	Forward	GAAGTTTTTGAAGAGGGCTGAGA
	Reverse	TGCTTGAAGTTTCACTGGCATC
U6	Forward	CTCGCTTCGGCAGCACA
	Reverse	AACGCTTCACGAATTTGCGT
HPRT1	Forward	TGACACTGGCAAAACAATGCA
	Reverse	GGTCCTTTTCACCAGCAAGCT

### Inflammatory factor detection

We measured inflammatory mediator levels (IL-6, TNF-α, and IL-8) in cell culture supernatants using commercial ELISA kits (Thermo Fisher, USA). All experimental procedures were performed according to the manufacturer’s instructions, with absorbance measurements conducted at 450 nm on a microplate reader for subsequent cytokine concentration calculations based on standard curves.

### Dual-luciferase reporter

Based on TargetScan (http://www.targetscan.org) prediction of miR-142-5p and *CXCL8* binding sites, we built the wild-type and mutant pGL3-Control carrier (Promega, USA). After the vectors were co-transfected with miR-142-5p mimics or NC respectively, they were cultured for 48 h. Finally, the dual-luciferase reporter gene assay system (Yeasen, China) was used to quantitatively analyze the luciferase activity.

### Western blot analysis

Cells were lysed using RIPA lysis buffer (Beyotime, China) containing protease and phosphatase inhibitors (Beyotime, China) after LPS treatment. Following 30-minute ice incubation, the lysates were centrifuged to collect supernatants. Protein concentrations were determined by BCA assay (Thermo Fisher, USA), and samples were mixed with 5×SDS loading buffer (Beyotime, China) and boiled for denaturation. Equal amounts of protein samples were separated by SDS-PAGE (15% separating gel, Bio-Rad, USA) at 120 V, then transferred to PVDF membranes (Millipore, USA) using wet transfer method. After blocking with 5% skim milk for 1 h, membranes were incubated sequentially with anti-*CXCL8* primary antibody (Abcam, UK, 1:1000) and HRP-conjugated secondary antibody (CST, USA, 1:5000). Protein bands were visualized using ECL chemiluminescent reagent (Millipore, USA) and captured by a chemiluminescence imaging system (Bio-Rad, USA). Band intensities were quantified using ImageJ software (NIH, USA) with GAPDH as internal control for relative expression calculation.

### Statistical analysis

In this study, data analysis was conducted using two statistical Software, SPSS 23.0 (from IBM) and GraphPad Prism 6.0 (from GraphPad Software). All data in this study came from three separate experimental replicates. Results are presented as mean values with standard deviations (mean ± SD). The analysis of differences between groups was conducted using the independent sample t-test (comparison between two groups) or one-way analysis of variance (comparison among multiple groups). Pearson/Spearman correlation tests assessed relationships between miR-142-5p levels and clinical parameters. ROC analysis revealed miR-142-5p’s diagnostic potential for sepsis (AUC > 0.7). Multivariate logistic regression analysis showed independent risk factors for sepsis (adjusted OR, 95% CI). Statistical tests were conducted on both sides, and the significance level was set as *P* < 0.05.

## Results

### Demographic and clinical characteristics of patients with sepsis and healthy controls

The study population consisted of 110 controls (58 male, 52 female; mean age 52.60 ± 7.05 years) and 110 sepsis patients (65 male, 45 female; mean age 53.48 ± 8.92 years). The baseline demographic and clinical characteristics (age, sex, and BMI) were comparable across all study groups, with no significant differences observed (*P* > 0.05, Table 1).

However, sepsis patients exhibited significantly lower albumin levels (27.65 ± 5.92 vs. 40.82 ± 4.35 g/L) and markedly elevated inflammatory markers including WBC count (13.82 ± 3.81 vs. 6.16 ± 1.35 × 10⁹/L), CRP (72.19 ± 20.17 vs. 5.89 ± 1.87 mg/L), and PCT (6.88 ± 3.40 vs. 0.05 ± 0.03 ng/mL) compared to healthy controls (all *P* < 0.001, Table 1). Additionally, sepsis patients showed significantly higher serum creatinine levels (128.72 ± 32.91 vs. 76.55 ± 18.69 µmol/L, *P* < 0.001) and a mean APACHE II score of 13.73 ± 5.17.

### miR-142-5p has the dual potential as both a diagnostic marker and a therapeutic target for sepsis

**Fig. 1:**
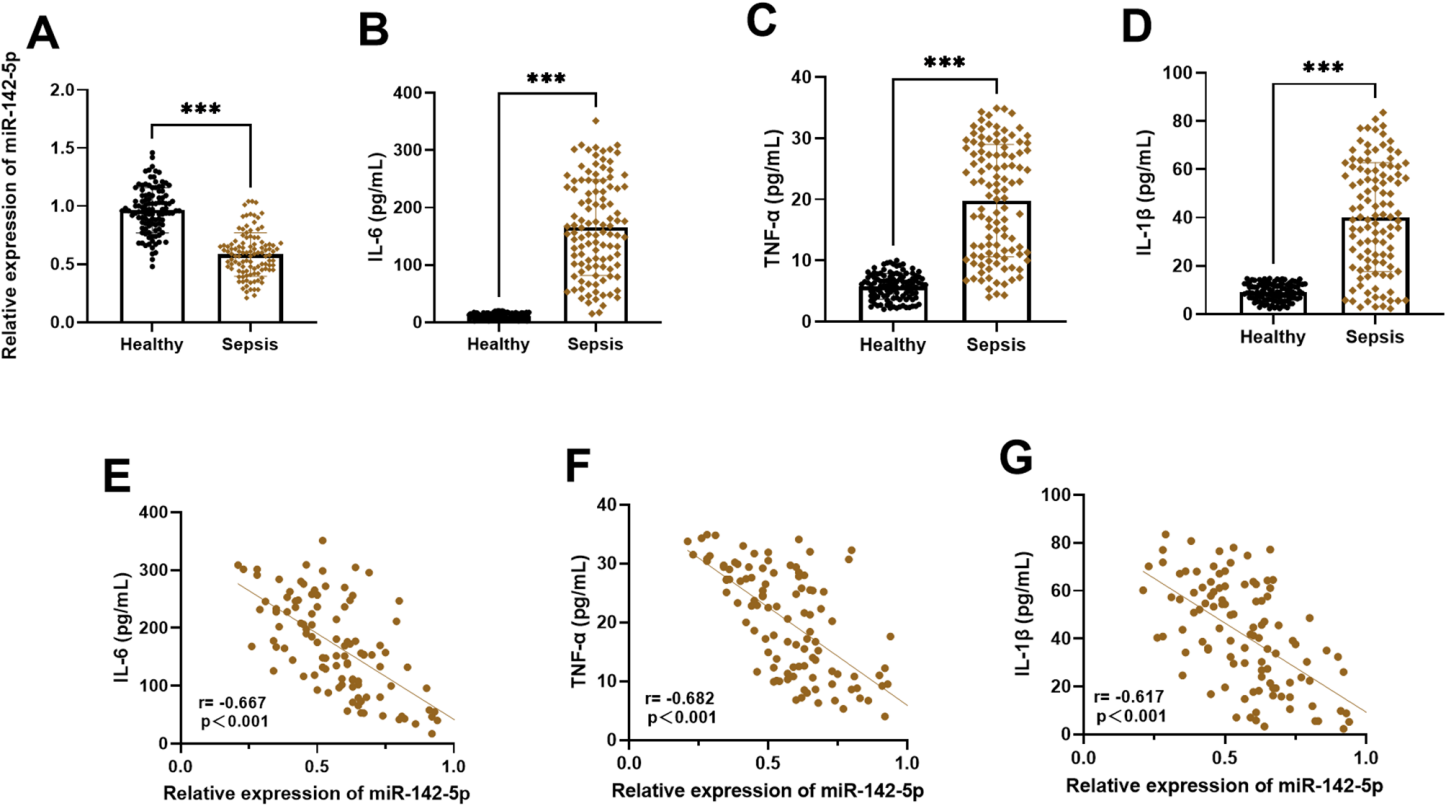
Analysis of miR-142-5p expression and inflammatory markers in sepsis patients. *** *P* < 0.001. (**A**) Serum miR-142-5p expression levels in sepsis patients compared to healthy controls. (**B**-**D**) The concentrations of IL-6 (**B**), TNF-α (**C**) and IL-1β (**D**) in each study group. (**E**-**G**) Correlation analyses between serum miR-142-5p and IL-6 (**E**), TNF-α (**F**), or IL-1β (**G**)

**Fig. 2:**
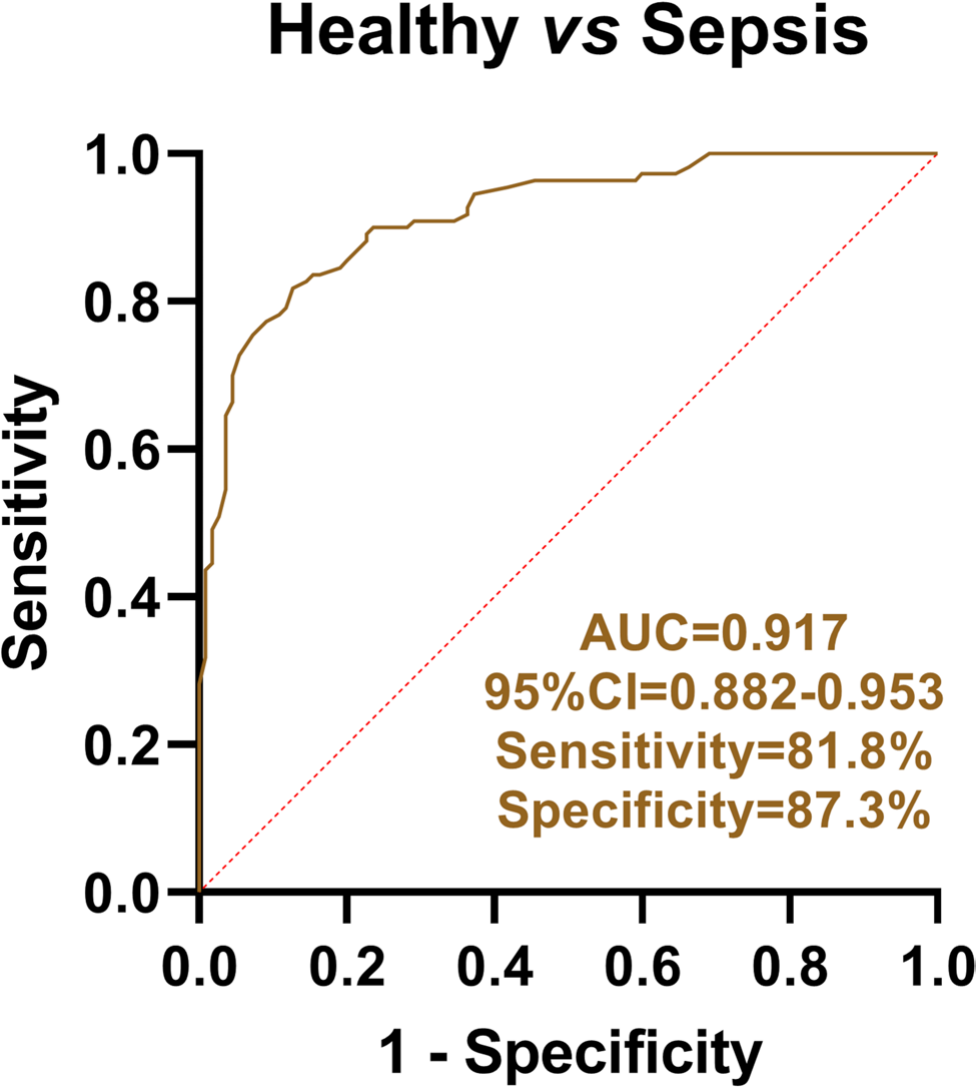
ROC curve analysis of serum miR-142-5p in the diagnosis of sepsis

We quantitatively analyzed serum concentrations of miR-142-5p and pro-inflammatory cytokines (IL-6, TNF-α, IL-1β) in both sepsis patients and healthy controls. Our findings revealed significantly reduced serum miR-142-5p levels in sepsis patients versus healthy controls (Fig. 1A). In contrast, key inflammatory markers (IL-6, TNF-α, IL-1β) showed substantial increases (Figs. 1B-D). Correlation analysis showed that miR-142-5p was significantly negatively correlated with these inflammatory factors (Figs. 1E-G; IL-6: *r* = -0.667, *P* < 0.001; TNF-α: *r* = -0.662, *P* < 0.001; IL-1β: *r* = -0.617, *P* < 0.001). Further ROC curve analysis was performed, and the results are shown in Fig. 2. The AUC of miR-142-5p in the diagnosis of sepsis reached 0.917 (0.882–0.953), and the sensitivity and specificity were 81.8% and 87.3%, respectively. Our results suggest that miR-142-5p may serve not only as a potential biomarker but also as a possible regulatory factor in sepsis, where its reduced expression could promote increased proinflammatory cytokine release, potentially exacerbating inflammatory responses under septic conditions.

### The expression levels of miR-142-5p demonstrated significant correlations with key clinical indicators in sepsis patients

The correlation between miR-142-5p expression levels and key clinical indicators in sepsis patients was analyzed, with results shown in Table 3. miR-142-5p was positively correlated with serum albumin at a moderate intensity (*r* = 0.588, *P* < 0.001). Conversely, miR-142-5p showed strong negative correlations with inflammatory markers including WBC count (*r* = -0.627), CRP (*r* = -0.707), and PCT (*r* = -0.611), as well as disease severity assessed by APACHE II score (*r* = -0.696), all with *P* < 0.001. miR-142-5p was moderately negatively correlated with serum creatinine (*r* = -0.564, *P* < 0.001). These findings suggest that higher miR-142-5p expression is associated with improved clinical outcomes, particularly showing the strongest inverse relationships with CRP and APACHE II score, indicating its potential as a biomarker for sepsis severity and systemic inflammation. All correlations were highly significant (*P* < 0.001), encompassing WBC count, CRP, PCT, and APACHE II scores.

**Table 3:** The correlation between the expression of miR-142-5p and clinical indicators in sepsis patients

Parameters	miR-142-5p expression	
	Correlation coefficient (*r*)	*P* value
Albumin	0.588	<0.001
WBC	-0.627	<0.001
Scr	-0.564	<0.001
CRP	-0.707	<0.001
PCT	-0.611	<0.001
APACHE II score	-0.696	<0.001

WBC, white blood cells; Scr, serum creatinine; CRP, C-reactive protein; PCT, procalcitonin; APACHE II, acute pathology and chronic health evaluation II

### Sepsis-related risk factors

Logistic regression revealed multiple significant predictors of sepsis risk (Table 4), including both protective and contributing factors. Elevated levels of inflammatory markers are significantly associated with the risk of sepsis: CRP (HR = 1.878, 95% CI 1.040–3.390, *P* = 0.037) and PCT (HR = 1.833, 95% CI 1.021–3.291, *P* = 0.042) For each one-unit increase, the risk of sepsis increased by 87.8% and 83.3% respectively. Notably, higher expression of miR-142-5p emerged as a significant protective factor (HR = 0.498, 95% CI 0.282–0.882, *P* = 0.017), indicating a 50.2% risk reduction per unit increase in expression. Demographic characteristics (age, sex), metabolic parameters (BMI, albumin), and other laboratory indices (WBC, serum creatinine) did not reach statistical significance (all *P* > 0.05), although white blood cell count (HR = 1.504) and serum creatinine (HR = 1.466) showed clinically meaningful trends. These findings highlight CRP and PCT as sensitive early warning indicators for sepsis, while suggesting miR-142-5p could represent a candidate therapeutic target for sepsis, pending validation in preclinical animal models and studies of delivery mechanisms.

**Table 4:** Logistic regression analysis of risk factors associated with sepsis

Variable	HR	95% CI for HR		*P* value
		Lower	Upper	
Age	1.371	0.774	2.430	0.280
Sex	1.071	0.596	1.924	0.819
BMI	0.888	0.503	1.567	0.682
Albumin	1.412	0.787	2.531	0.247
WBC	1.504	0.831	2.724	0.177
Scr	1.466	0.828	2.595	0.190
CRP	1.878	1.040	3.390	0.037*
PCT	1.833	1.021	3.291	0.042*
miR-142-5p	0.498	0.282	0.882	0.017*

BMI, body mass index; Scr, serum creatinine; WBC, white blood cells, CRP, C-reactive protein; PCT, procalcitonin. * *P* <0.05

### miR-142-5p suppresses cellular inflammatory responses

**Fig. 3:**
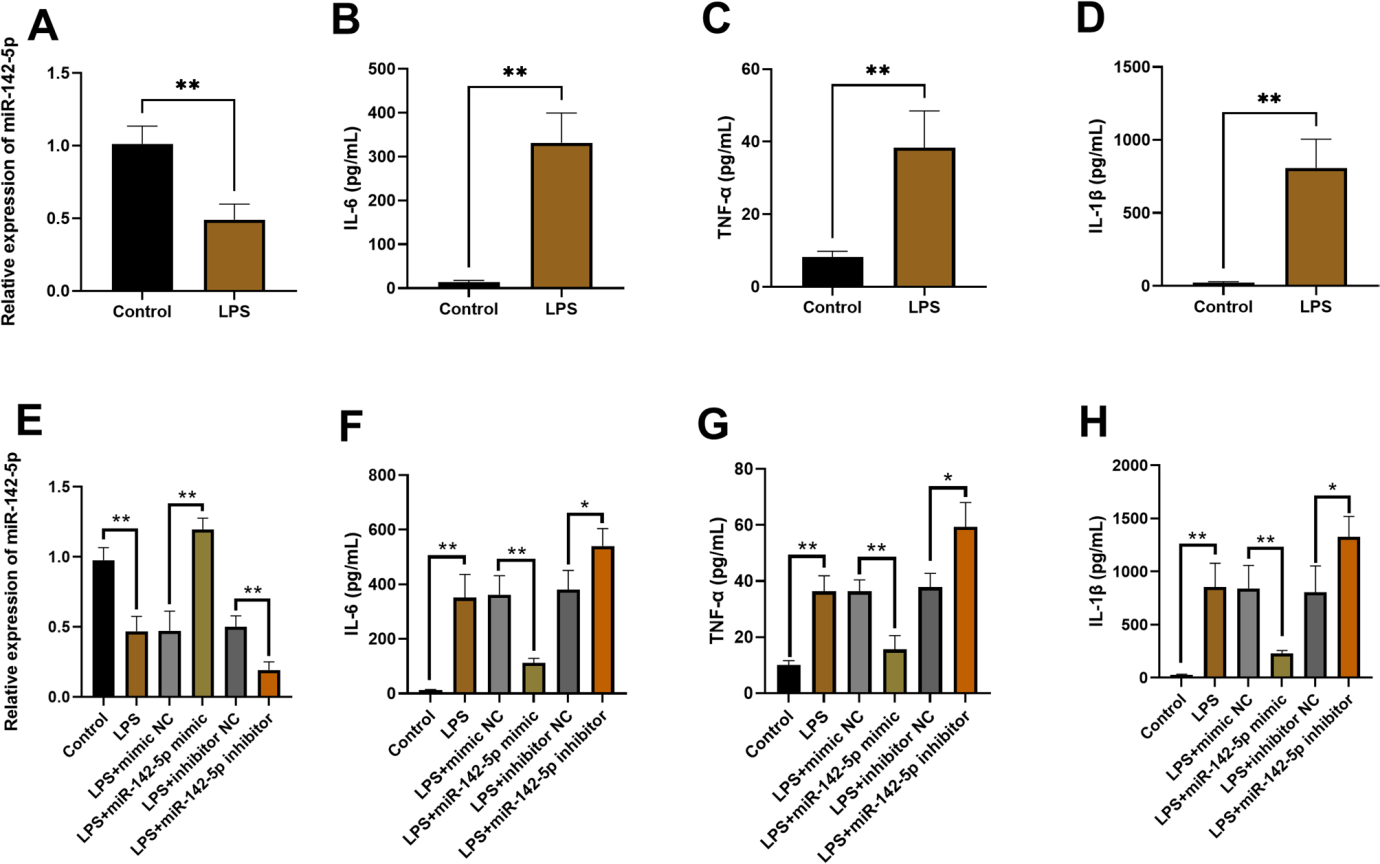
Effects of induction and transfection on miR-142-5p expression and inflammatory markers. *P < 0.05, **P < 0.01. (**A**) miR-142-5p expression levels in cells post-induction. (**B**-**D**) Levels of inflammatory cytokines (IL-6, TNF-α, IL-1β) in cells after induction. (**E**) miR-142-5p expression in cells post-transfection. (**F**-**H**) The expression levels of IL-6/TNF-α/IL-1β in transfected cells

To investigate miR-142-5p’s immunomodulatory role, we employed an LPS-stimulated THP-1 cell model combined with gain/loss-of-function experiments to systematically assess its effects on cytokine production. LPS challenge triggered reciprocal regulation: miR-142-5p expression decreased significantly (Fig. 3A) while pro-inflammatory cytokines (IL-6, TNF-α, IL-1β) showed substantial elevation (Figs. 3B-D). These results indicate that LPS successfully induced a sepsis-related inflammatory response, characterized by suppressed miR-142-5p expression and excessive release of pro-inflammatory cytokines (IL-6, TNF-α, IL-1β).

Based on these findings, we performed transfection experiments using miR-142-5p mimic and miR-142-5p inhibitor. Compared with the mimic NC group, miR-142-5p mimic transfection significantly upregulated miR-142-5p expression, confirming successful enhancement of intracellular miR-142-5p levels. Compared with the control group or the inhibitor negative control group, the expression level of miR-142-5p in the miR-142-5p inhibitor group was significantly decreased (Fig. 3E), indicating that its expression was effectively inhibited. These experiments established a reliable model for further investigation of miR-142-5p’s regulatory mechanisms.

Using this model, we examined miR-142-5p’s regulation of cytokine production. miR-142-5p overexpression (mimics) significantly reduced IL-6, TNF-α, and IL-1β secretion (*P* < 0.01, Fig. 3F-H), while inhibition increased their release (*P* < 0.05). These contrasting effects establish miR-142-5p as a potent negative regulator of inflammation, with expression levels directly controlling cytokine output.

*miR-142-5p negatively regulates CXCL8 through targeted inhibition.*

**Fig. 4:**
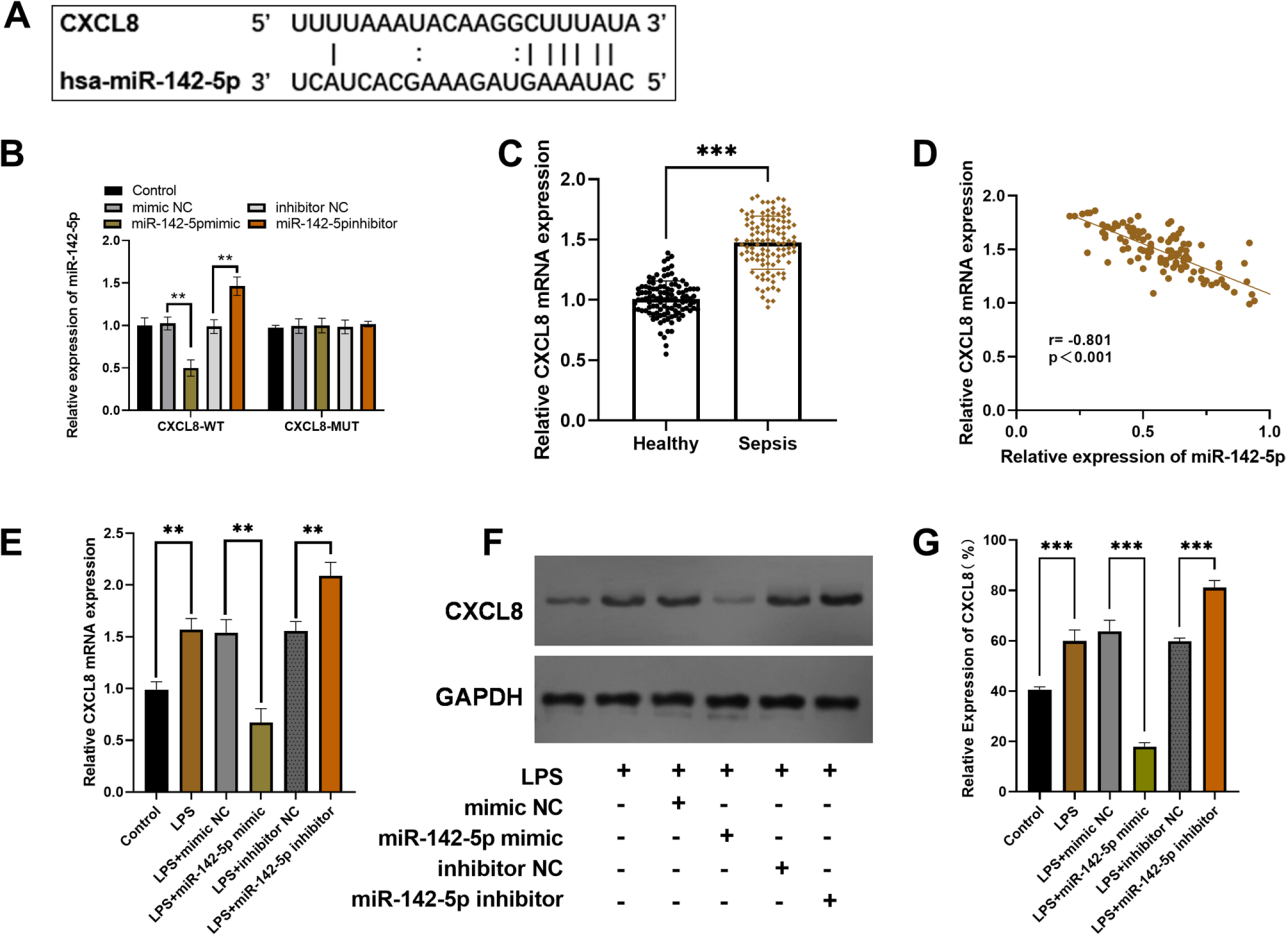
Mechanistic studies of miR-142-5p and *CXCL8* interaction. **P < 0.01, *** *P* < 0.001. (**A**) miR-142-5p binding sites in the 3’-UTR of *CXCL8* mRNA. (**B**) Luciferase verification of miR-142-5p targeting *CXCL8*. (**C**) Serum *CXCL8* mRNA expression levels in patient cohorts. (**D**) The expression correlation between miR-142-5p and *CXCL8*. (**E**) Relative expression levels of *CXCL8* mRNA under miR-142-5p mimic or inhibition conditions. (**F**) Western blot analysis of CXCL8 protein expression in transfected cells under miR-142-5p regulation. (**G**) Quantitative analysis of CXCL8 protein expression levels under miR-142-5p regulation

TargetScan predicts that miR-142-5p can directly target *CXCL8* (the binding site is shown in Fig. 4A). Dual-luciferase assays validated this interaction, with miR-142-5p mimics reducing wild-type *CXCL8* luciferase activity (*P* < 0.01) while showing no effect on mutant constructs (Fig. 4B). Clinical data revealed elevated *CXCL8* mRNA levels in sepsis patients versus controls (Fig. 4C), which inversely correlated with miR-142-5p expression (*r* = -0.801, *P* < 0.001, Fig. 4D). Combined qPCR and Western blot analyses demonstrated that LPS stimulation significantly upregulated *CXCL8* expression at both transcriptional and translational levels (*P* < 0.01), establishing a robust inflammatory model. Functional studies revealed that miR-142-5p overexpression potently suppressed LPS-induced *CXCL8* expression, whereas miR-142-5p inhibition conversely enhanced *CXCL8* production (*P* < 0.01, Fig. 4E-G), establishing miR-142-5p as a negative regulator of *CXCL8*.

### miR-142-5p mediates inflammatory responses through regulation of CXCL8 expression

**Fig. 5:**
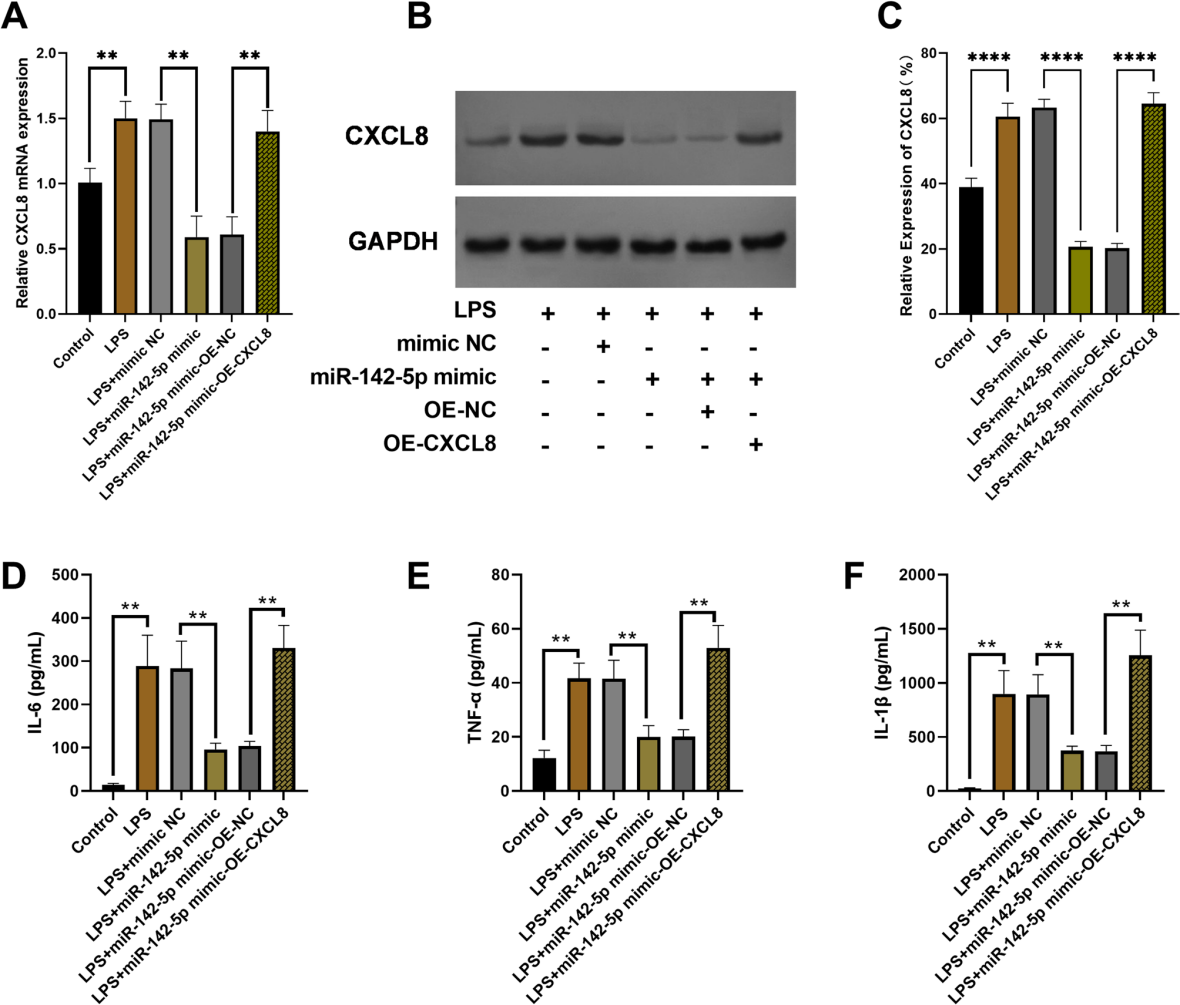
Functional rescue experiments validating the miR-142-5p/*CXCL8* regulatory axis in inflammatory responses. **P < 0.01, *** *P* < 0.001. (**A**) Relative expression levels of *CXCL8* mRNA in rescue experiments. (**B**) Western blot analysis of CXCL8 protein expression in transfected cells during rescue experiments. (**C**) Quantitative analysis of CXCL8 protein expression levels in rescue experiments. (**D**-**F**) The expression profiles of IL-6/TNF-α/IL-1β in transfected and co-transfected cells

LPS induction and co-transfection experiments systematically elucidated the regulatory mechanism of miR-142-5p on *CXCL8* and its role in inflammatory responses. As shown in Figs. 5A-C, LPS stimulation significantly upregulated *CXCL8* expression (*P* < 0.01), successfully establishing an inflammatory model. miR-142-5p overexpression (LPS + miR-142-5p mimic group) significantly suppressed *CXCL8* expression at both mRNA and protein levels (*P* < 0.01). Functional rescue experiments demonstrated significant recovery of *CXCL8* expression in the LPS + mimic + OE-*CXCL8* group (*P* < 0.01). These results confirm the direct inhibitory effect of miR-142-5p on *CXCL8* and molecularly define their regulatory relationship through rescue experiments, providing crucial evidence for understanding inflammatory response mechanisms.

Using this validated model, we demonstrated that miR-142-5p counteracts LPS-induced inflammation, with miR-142-5p mimic transfection significantly attenuating the elevated expression of IL-6, TNF-α and IL-1β caused by LPS stimulation. Notably, co-transfection with OE-*CXCL8* led to varying degrees of recovery in the expression levels of all three inflammatory factors (Figs. 5D-F). These results not only confirm the anti-inflammatory effect of miR-142-5p but also directly demonstrate that *CXCL8* serves as its critical downstream target for mediating anti-inflammatory effects.

## Discussion

The pathogenesis of sepsis stems from the host’s dysregulated immune response to infection, which triggers massive release of pro-inflammatory factors in the early stage, leading to multiple organ dysfunction syndrome (MODS) [19]. Growing evidence suggests that early screening and diagnosis of sepsis can significantly improve cure rates. Current gold-standard biomarkers (PCT and CRP) demonstrate limited diagnostic accuracy for sepsis, exhibiting insufficient sensitivity and specificity in clinical practice [20]. Therefore, identifying novel diagnostic markers and therapeutic targets holds significant importance for global healthcare systems.

Emerging studies indicate that differentially expressed miRNAs play crucial roles in sepsis through gene regulation, serving not only as novel diagnostic biomarkers but also as potential therapeutic targets [21]. In clinical diagnostics, miRNAs such as miR-150 [22], miR-206 [23] and miR-147b [24] have been identified as potential diagnostic markers for sepsis. Therapeutically, miR-150-5p has shown promise as a treatment target for sepsis-induced myocardial depression [25]. miR-142-5p is a specific predictor of acute kidney injury in elderly patients with sepsis [26]. Previous studies demonstrate that miR-142-5p ameliorates sepsis-associated lung injury through *PTEN* downregulation and subsequent PI3K/Akt pathway activation [27]. Sepsis patients showed significantly depressed serum miR-142-5p levels versus controls, with upregulated IL-6, TNF-α and IL-1β. ROC analysis and multivariate regression confirmed miR-142-5p’s diagnostic value, supporting its dual role as both a sepsis biomarker and inflammatory regulator.

In studies of sepsis pathogenesis, inflammatory cytokines (IL-6, TNF-α, IL-1β) serve not only as mediators of systemic inflammatory response but also as crucial molecular markers for diagnosis, prognosis evaluation, and treatment of sepsis [28]. For instance, in neonatal sepsis research, these inflammatory factors function as mediators of inflammation and can be utilized for diagnosis and therapeutic efficacy assessment [29, 30]. IL-6 (interleukin-6), a pivotal inflammatory mediator, promotes acute-phase protein release and amplifies inflammatory responses, making it a valuable biomarker for sepsis diagnosis and prognosis [31]. In early sepsis, TNF-α emerges as the predominant inflammatory mediator by initiating NF-κB signaling and promoting secondary cytokine release, thereby driving systemic inflammation [32]. IL-1β, a potent pro-inflammatory cytokine with essential immunological functions, may exacerbate tissue damage under pathological conditions [33, 34]. To assess disease severity and prognostic value, we quantified serum levels of key pro-inflammatory cytokines (IL-6, TNF-α, IL-1β) in sepsis patients. Complementary in vitro studies using LPS-stimulated THP-1 macrophages revealed a strong inverse correlation (all *P* < 0.01) between miR-142-5p expression and secretion of these inflammatory mediators, suggesting miR-142-5p’s role in modulating cytokine storms. Notably, inhibition of miR-142-5p expression led to markedly increased secretion of inflammatory factors, while miR-142-5p overexpression effectively suppressed their release. These findings suggest that miR-142-5p may play a pivotal role in regulating inflammatory responses during sepsis, where its downregulation potentially exacerbates inflammation, whereas restoring or enhancing its expression could represent a promising therapeutic strategy for mitigating sepsis-associated inflammation.

Extensive research has established that NF-κB pathway activation directly upregulates TNF-α and IL-6 gene transcription through specific κB-site binding in their promoter regions [35], while NLRP3 inflammasome activation is closely associated with the maturation and secretion of IL-1β [36]. Therefore, miR-142-5p may influence the release of TNF-α, IL-6, and IL-1β by regulating inflammatory signaling pathways (such as NF-κB or the NLRP3 inflammasome). However, whether miR-142-5p can directly regulate pro-inflammatory cytokines in sepsis remains unclear.

*CXCL8* encodes the pro-inflammatory cytokine interleukin-8 (IL-8) [14]. Emerging evidence demonstrates that *CXCL8* is significantly upregulated in patients with severe sepsis/septic shock, and its expression level positively correlates with disease severity, suggesting its potential as a biomarker for assessing sepsis progression [37]. Mechanistically, *CXCL8* is not only regulated by inflammatory cytokines like IL-1β but also reciprocally modulates both NF-κB and MAPK signaling pathways [38, 39]. These signaling pathways play a key role in the pathogenesis of sepsis and are closely related to the release of various inflammatory mediators, such as IL-6, TNF-α [2]. Thus, *CXCL8* may exacerbate the inflammatory cascade in sepsis by amplifying these critical signaling pathways.

We found that miR-142-5p suppresses *CXCL8*, consistent with evidence that modulating membrane signaling dampens inflammatory cues. Local anesthetics (e.g., lidocaine) have been shown to reduce cytokine storms by blocking voltage-gated Na⁺ channels [40, 41], mirroring miR-142-5p’s throttling effect on *CXCL8*. Notably, Nav channel inhibition in cancer models similarly attenuates IL-8-driven cell migration [42, 43], suggesting a conserved mechanism across inflammatory contexts.

The *CXCL8*-mediated leukocyte trafficking in sepsis shares mechanistic parallels with Helicobacter pylori infection, where bacterial products directly upregulate *CXCL8* to drive gastric epithelial cell migration [44]. Although sepsis is not localized to the gut, this host-pathogen crosstalk exemplifies how *CXCL8* amplification can override normal chemotaxis checkpoints–a pathway similarly disrupted in our LPS model. Targeting this axis with miR-142-5p may thus offer broader anti-adhesion benefits beyond cytokine suppression.

Bioinformatic prediction (TargetScan) and functional validation (dual-luciferase assays) confirmed miR-142-5p’s direct binding to *CXCL8* ‘s 3’UTR, demonstrating its repressive effect on *CXCL8* expression. This discovery provides novel molecular insights into the regulatory mechanisms of inflammatory responses in sepsis. Experiments have confirmed that miR-142-5p significantly reduces the release of IL-6/TNF-α/IL-1β by targeting and inhibiting *CXCL8*. In the LPS-stimulated inflammatory model, miR-142-5p overexpression significantly suppressed pro-inflammatory cytokine production (IL-6, TNF-α, IL-1β). Notably, simultaneous *CXCL8* overexpression partially reversed the suppressive effect of miR-142-5p, consistent with *CXCL8* functioning downstream of miR-142-5p in this experimental system. These data support the anti-inflammatory role of miR-142-5p and suggest *CXCL8* may contribute to its regulatory effects, though additional targets likely participate in this process.

Collectively, this study identifies miR-142-5p as a novel regulator of *CXCL8*-mediated inflammation in sepsis, adding to the existing knowledge on miRNA-based immunomodulation in this condition. While our findings robustly support *CXCL8*’s role in miR-142-5p-mediated effects, we acknowledge certain limitations. As demonstrated in Liu et al.‘s study [45] using CRISPR screening and cell line IC50 data to reveal novel key genes in trametinib resistance, genome-wide CRISPR knockout or activation screening could systematically map both upstream regulators and downstream effectors of the *CXCL8* axis, potentially uncovering additional layers of this regulatory network. Future clinical trials could explore synergies between miR-142-5p mimics and traditional Chinese medicine (TCM) immunomodulators, which have demonstrated efficacy in curbing hyperinflammation during liver transplantation [46] and H. pylori infection [47]. Additionally, future studies employing CRISPR-based approaches could further elucidate the upstream and downstream regulatory networks of the *CXCL8* axis, as demonstrated in recent functional genomic screens for drug resistance mechanisms, thereby expanding the mechanistic depth of the current findings. Future studies should further explore the specific regulatory patterns of the miR-142-5p/*CXCL8* axis in different sepsis subtypes through integration of pan-cancer analyses of cuproptosis [48] and disulfidptosis [49], while generative adversarial networks (GANs) applied to miR-142-5p expression profiling [50] could identify subtype-specific therapeutic windows, particularly in sepsis with distinct metabolic or DNA damage signatures [18], ultimately advancing its potential clinical applications as a therapeutic target.

## Data Availability

The datasets used and/or analysed during the current study are available from the corresponding author on reasonable request.
